# Repeated COVID-19 Vaccination Drives Memory T- and B-cell Responses in Kidney Transplant Recipients: Results From a Multicenter Randomized Controlled Trial

**DOI:** 10.1097/TP.0000000000005119

**Published:** 2024-11-21

**Authors:** S. Reshwan K. Malahe, Yvette den Hartog, Wim J. R. Rietdijk, Debbie van Baarle, Ronella de Kuiper, Derek Reijerkerk, Alicia M. Ras, Daryl Geers, Dimitri A. Diavatopoulos, A. Lianne Messchendorp, Renate G. van der Molen, Céline Imhof, Sophie C. Frölke, Frederike J. Bemelman, Ron T. Gansevoort, Luuk B. Hilbrands, Jan-Stephan F. Sanders, Corine H. GeurtsvanKessel, Marcia M. L. Kho, Rory D. de Vries, Marlies E. J. Reinders, Carla C. Baan

**Affiliations:** 1 Department of Internal Medicine, Nephrology and Transplantation, Erasmus MC Transplant Institute, Erasmus University Medical Center, Rotterdam, the Netherlands.; 2 Department of Hospital Pharmacy, Erasmus University Medical Center, Rotterdam, the Netherlands.; 3 Department of Medical Microbiology and Infection Prevention, Virology and Immunology Research Group, University Medical Center Groningen, Groningen, the Netherlands.; 4 Center for Infectious Disease Control, National Institute for Public Health and the Environment, Bilthoven, the Netherlands.; 5 Department of Viroscience, Erasmus University Medical Center, Rotterdam, the Netherlands.; 6 Radboud Institute for Molecular Life Sciences, Department of Laboratory Medicine, Laboratory of Medical Immunology, Radboud University Medical Center Nijmegen, Nijmegen, the Netherlands.; 7 Radboud Center for Infectious Diseases, Radboud University Medical Center Nijmegen, Nijmegen, the Netherlands.; 8 Department of Internal Medicine, Division of Nephrology, University of Groningen, University Medical Center Groningen, Groningen, the Netherlands.; 9 Department of Experimental Immunology, Amsterdam Infection and Immunity Institute, Amsterdam UMC, University of Amsterdam, Amsterdam, the Netherlands.; 10 Renal Transplant Unit, Amsterdam UMC, University of Amsterdam, Amsterdam, the Netherlands.; 11 Department of Nephrology, Radboud Institute for Health Sciences, Radboud University Medical Center, Nijmegen, the Netherlands.; (Department of Nephrology and Hypertension, University Medical Center Utrecht, Utrecht, the Netherlands); (Department of Nephrology, Radboud University Medical Center, Radboud Institute for Health Sciences, Nijmegen, the Netherlands); (Department of Internal Medicine, Division of Nephrology, Maastricht University Medical Center and CARIM School for Cardiovascular Disease, University of Maastricht, Maastricht, the Netherlands); (Dutch Registry RENINE, Nefrovisie, Utrecht, the Netherlands); (Department Viroscience, Erasmus Medical Center, Rotterdam, the Netherlands); (Renal Transplant Unit, Amsterdam UMC, University of Amsterdam, Amsterdam, the Netherlands); (Department of Medical Microbiology and Infection Prevention, University Medical Center Groningen, Groningen, the Netherlands); (Department of Internal Medicine, Division of Nephrology, University of Groningen, University Medical Center Groningen, Groningen, the Netherlands); (Department of Internal Medicine, Nephrology and Transplantation, Erasmus MC Transplant Institute, Erasmus Medical Center, Rotterdam, the Netherlands); (Department of Nephrology, Radboud University Medical Center, Radboud Institute for Health Sciences, Nijmegen, the Netherlands); (Department of Nephrology, Radboud University Medical Center, Radboud Institute for Health Sciences, Nijmegen, the Netherlands); (Department Viroscience, Erasmus Medical Center, Rotterdam, the Netherlands); (Department Viroscience, Erasmus Medical Center, Rotterdam, the Netherlands); (Department Viroscience, Erasmus Medical Center, Rotterdam, the Netherlands); (Center for Infectious Disease Control, National Institute for Public Health and the Environment, Bilthoven, the Netherlands); (Department of Internal Medicine, Division of Nephrology, Maastricht University Medical Center and CARIM School for Cardiovascular Disease, University of Maastricht, Maastricht, the Netherlands); (Department of Nephrology, Leiden University Medical Center, Leiden, the Netherlands)

## Abstract

**Background.:**

Insight into cellular immune responses to COVID-19 vaccinations is crucial for optimizing booster programs in kidney transplant recipients (KTRs).

**Methods.:**

In an immunologic substudy of a multicenter randomized controlled trial (NCT05030974) investigating different repeated vaccination strategies in KTR who showed poor serological responses after 2 or 3 doses of an messenger RNA (mRNA)-based vaccine, we compared SARS-CoV-2-specific interleukin-21 memory T-cell and B-cell responses by enzyme-linked immunosorbent spot (ELISpot) assays and serum IgG antibody levels. Patients were randomized to receive: a single dose of mRNA-1273 (100 μg, n = 25), a double dose of mRNA-1273 (2 × 100 μg, n = 25), or a single dose of adenovirus type 26 encoding the SARS-CoV-2 spike glycoprotein (Ad26.COV2.S) (n = 25). In parallel, we also examined responses in 50 KTR receiving 100 μg mRNA-1273, randomized to continue (n = 25) or discontinue (n = 25) mycophenolate mofetil/mycophenolic acid. As a reference, the data were compared with KTR who received 2 primary mRNA-1273 vaccinations.

**Results.:**

Repeated vaccination increased the seroconversion rate from 21% to 66% in all patients, which was strongly associated with enhanced levels of SARS-CoV-2-specific interleukin-21 memory T cells (odd ratio, 3.84 [1.89-7.78]; *P* < 0.001) and B cells (odd ratio, 35.93 [6.94-186.04]; *P* < 0.001). There were no significant differences observed in these responses among various vaccination strategies. In contrast to KTR vaccinated with 2 primary vaccinations, the number of antigen-specific memory B cells demonstrated potential for classifying seroconversion after repeated vaccination (area under the curve, 0.64; 95% confidence interval, 0.37-0.90; *P* = 0.26 and area under the curve, 0.95; confidence interval, 0.87-0.97; *P* < 0.0001, respectively).

**Conclusions.:**

Our study emphasizes the importance of virus-specific memory T- and B-cell responses for comprehensive understanding of COVID-19 vaccine efficacy among KTR.

## INTRODUCTION

Compared with healthy individuals, kidney transplant recipients (KTRs) show lower antibody levels after vaccination against hepatitis B virus, influenza virus, *Streptococcus pneumoniae*, and COVID-19 because of their kidney disease and the use of immunosuppressive drugs.^1-3^ COVID-19 vaccine-induced antibody responses remain suboptimal, even after repeated vaccination, leaving KTR at increased risk for COVID-19-associated morbidity and mortality.^1,4^ Alternative vaccination regimens have been explored to optimize protection against COVID-19 for these high-risk patients.^5^ Although antibody response is typically the primary immunologic outcome in clinical trials evaluating novel COVID-19 vaccines or vaccination strategies, cellular immune responses likely represent an important mechanism of protection, including memory T and B cells.^6,7^ This is particularly relevant for immunocompromised patients, in whom cellular immunes responses to COVID-19 vaccination may be discordant from humoral immune responses that are strongly impaired.^8^ Clinical data suggests that T-cell memory may represent a potential immunologic correlate of protection against severe COVID-19 in patients with impaired B-cell–mediated immune responses, such as KTR.^9-13^ A recent study illustrated that vaccination reduced the risk of severe COVID-19 in B-cell deficient individuals, implying a potentially protective role for SARS-CoV-2-specific T cells in this patient population.^14^ Additionally, COVID-19-associated morbidity and mortality in KTR has reduced since the start of the vaccination campaigns, despite multiple studies reporting impaired antibody responses in these patients.^1,3,9-13^

Interleukin-21 (IL-21) is a cytokine that plays a crucial role in T-cell–dependent B-cell activation within germinal centers. It contributes significantly to the development of virus-specific memory B cells and antibodies, and increases the cytotoxic activity of natural killer cells and CD8^+^ cytotoxic T cells.^15-19^ Therefore, analysis of IL-21–mediated immune responses after vaccination could contribute to a better understanding of cellular memory immune responses encompassing both memory T- and B-cell responses.

In this study, we investigated whether KTR, who initially had a poor serological response to messenger RNA (mRNA)-based vaccinations, developed cellular immune responses after repeated COVID-19 vaccination via different vaccination strategies. We analyzed SARS-CoV-2 spike-specific IL-21 and interferon gamma (IFN-γ) producing memory T cells, memory B cells, and serological responses in patients who received one of 3 alternative revaccination regimens compared with those who received a control single-dose mRNA-1273 vaccination.

## MATERIALS AND METHODS

### Study Design

This is a post hoc immunologic analysis of a prospective, open-label, randomized, controlled trial (NCT05030974) as part of the repeated vaccination of the RECOVAC (Dutch Renal patients COVID-19 VACcination) consortium.^6^ Ethical approval was obtained from the Dutch Central Committee on Research Involving Human Subjects, the central ethics committee at the University Medical Center Groningen, and the local ethics committees of the participating centers.

### Patients

For this post hoc analysis, we utilized samples collected from KTR who participated in the RECOVAC repeated vaccination randomized controlled trial (NCT05030974), the results of which have been extensively described elsewhere.^6^ In short, the study included KTR who were classified as serological nonresponders after having received either 2 or 3 doses of an mRNA-based vaccine (defined as a SARS-CoV-2 spike protein [S1]-specific antibody response <10 binding antibody units [BAU]/mL 28 d after vaccination). Patients using any type of immunosuppressants were randomized to receive repeated vaccination with 100 μg mRNA-1273, 2 × 100 μg mRNA-1273, or adenovirus type 26 encoding the SARS-CoV-2 spike glycoprotein (Ad26.COV2.S) vaccination. In addition, KTR receiving triple immunosuppressive therapy, which includes calcineurin inhibitors, mycophenolate mofetil (MMF)/mycophenolic acid (MPA), and steroids, were randomized to either continue or discontinue MMF/MPA treatment for a duration of 2 wk, 1 wk before and 1 wk after receiving a repeated vaccination with 100 μg mRNA-1273. Serum samples and peripheral blood mononuclear cells (PBMCs) were collected at baseline and 28 d after repeated vaccination. At the baseline study measurement, it was observed that a proportion of patients had a low but measurable S1-specific antibody response. For this post hoc analysis, we randomly selected 125 KTR from the original pool of study participants (n = 333).^6^ Among the patients selected for in-depth analyses in this study, 42 of 125 patients had a S1-specific antibody response below the limit of detection (<10 BAU/mL) at baseline, and 83 of 125 had a measurable but low antibody response, with a maximum S1-specific IgG antibody level of 120 BAU/mL.

### Control Groups

As a reference for vaccine-induced immune responses post-repeated vaccination, we included previously published data on IL-21 memory T-cell, memory B-cell, and antibody responses from the RECOVAC Immune-response study, which investigated the safety and immunogenicity of primary vaccination with 2 doses of mRNA-1273.^8^ Specifically, we included results from healthy individuals without kidney disease (n = 47, estimated glomerular filtration rate >45 mL/min/1.73m^2^) and from KTR (n = 63, both with and without seroconversion).

### SARS-CoV-2-specific IL-21 Memory T-cell Response

The SARS-CoV-2-specific IL-21 memory T-cell response was measured at baseline and 28 d post-repeated vaccination by a commercially available IL-21 enzyme-linked immunosorbent spot (ELISpot), following the manufacturer’s guidelines (U-CyTech biosciences, Utrecht, the Netherlands).^8^ The assays were conducted in the same laboratory by the same technician. In short, PBMCs were isolated from heparinized blood using Ficoll Paque (GE Healthcare, Eindhoven, the Netherlands) and stored at –140 °C until analysis. Defrosted PBMCs were added to a 96-well polyvinylidene fluoride plate (300 000 PBMCs/well) precoated with an IL-21 capturing antibody. Subsequently, these cells were stimulated with SARS-CoV-2 antigens for 44 h, using a combination of S1 and S2 peptide pools (JPT Peptide Technologies, Berlin, Germany), consisting of 15-mer peptides with an 11-amino-acid overlap, encompassing the entire S1. IL-21–producing T cells were detected using a biotinylated detection antibody, streptavidin-horseradish peroxidase, and 3-Amino-9-ethylcarbazole. Finally, an ELISpot reader (Bioreader 6000-V; Bio-Sys, Karben, Germany) was used to count the spots. The IL-21 memory T-cell response was expressed as the number of SARS-CoV-2-specific IL-21–producing memory T cells (spots) per 1 million (10^6^) PBMCs. The lower limit of detection (LLoD) of this response was 3.3 IL-21 spots per 10^6^ PBMCs.

### SARS-CoV-2-specific Memory B-cell Response

The SARS-CoV-2-specific memory B-cell response was measured at baseline and 28 d post-repeated vaccination by a commercially available B-cell ELISpot following the instructions provided by the manufacturer (U-CyTech biosciences).^8^ The assays were conducted in the same laboratory by the same technician. Defrosted PBMCs were introduced to a 24-well plate (2 000 000 PBMCs/well) and polyclonally activated by preincubation with IL-2 and R848 for 7 d. Subsequently, cells (100 000 cells for SARS-CoV-2 antigen-specific and 10 000 cells for total IgG producing cells) were placed in a 96-well polyvinylidene fluoride plate, precoated with a capturing antibody for IgG, for 20 h. Biotinylated detection antibodies, including IgG Biotin to estimate total IgG producing cells and recombinant SARS-CoV-2 Spike His-tag Biotin Protein to estimate SARS-CoV-2 antigen-specific IgG producing cells, were added. Following this, horseradish peroxidase and 3-Amino-9-ethylcarbazole were used for staining, and spots were counted using an ELISpot reader (Bioreader 6000-V; Bio-Sys). The memory B-cell response was quantified as the number of SARS-CoV-2 antigen-specific IgG producing cells (spots) per 1 million (10^6^) PBMCs. The LLoD of this response was 10 B-cell spots per 10^6^ PBMCs.

### SARS-CoV-2 Spike S1-specific Antibody and IFN-γ Responses

SARS-CoV-2 spike S1-specific antibody and IFN-γ responses were previously assessed at baseline and 28 d after repeated vaccination and published in the context of the primary article of the randomized controlled trial.^6^ S1-specific antibodies were measured in serum by a validated fluorescent bead-based multiplex immunoassay as described previously.^20,21^ For the IFN-γ response, we used the measurements obtained by IFN-γ ELISpot assay for comparison since this was consistent with the ELISpot assays utilized for measuring IL-21 and B-cell responses.^22^ The LLoD of this response was 1.7 spots per 10^6^ PBMCs.

### Statistical Analysis

We analyzed the data in 4 steps. First, baseline characteristics for all 5 groups are described. Continuous data are expressed as median with interquartile range for non-normally distributed variables and as mean ± SD for normally distributed variables. Categorical data are presented as numbers and percentages. Second, we depicted immune responses as paired data, comparing baseline values with those observed 28 d after repeated vaccination. To compare the increase in responses among different groups, we determined the fold change per patient. This was calculated as the log-transformed response at 28 d after repeated vaccination divided by the log-transformed baseline response. We compared positive controls for both IL-21 and the B-cell ELISpot assay across the various groups. The Mann-Whitney *U* test, with pairwise deletion for missing data, was used for inter-group comparisons of medians. For within-group comparisons of medians, the Wilcoxon signed-rank test was used, utilizing listwise deletion for missing data. Third, we explored correlations between IL-21 memory T-cell and B-cell responses, IL-21 memory T-cell and antibody responses, memory B-cell and antibody responses, IL-21 memory T-cell and IFN-γ responses, and IFN-γ and antibody responses, all at 28 d after repeated vaccination. Spearman’s rank correlation coefficient (Spearman’s ρ) was used for these analyses, including all 5 groups together. Responders and nonresponders were classified using the LLoD for cellular responses and the cutoff value for IgG antibody levels, as described here earlier. Values were set at the LLoD (3.3 spots for IL-21 memory T-cell, 10 spots for memory B-cell, and 1.7 spots for IFN-γ response) in the case of 0 spots/10^6^ PBMCs at baseline and/or 28 d post-vaccination. Fourth, to determine the association between memory T- and B-cell activity and seroconversion, we compared data from these responses in healthy individuals and KTR following 2 primary mRNA-based vaccinations (previous study) with those in KTR following repeated vaccination.^8^ To achieve this, we initially categorized the correlation graphs between IL-21/memory B-cell responses and antibody response into 4 categories based on T-/B-cell response and serological responder status: (1) Absence of either T- or B-cell response with presence of seroconversion, (2) Presence of either T- or B-cell response with presence of seroconversion, (3) Absence of either T- or B-cell response with absence of seroconversion, and (4) Presence of either T- or B-cell response with absence of seroconversion. Furthermore, we generated receiver operating characteristic curves using IL-21 and memory B-cell responses as continuous variables and seroconversion (yes if ≥10 BAU/mL and no if <10 BAU/mL) as a binary outcome. We calculated the area under the curves (AUCs) along with 95% confidence intervals (CIs). Additionally, levels of IL-21–producing memory T cells and B cells were log-transformed and analyzed for their association with seroconversion using binary logistic regression, where estimates included odd ratios (ORs) with corresponding 95% CIs. Statistical analyses were conducted using SPSS (Version 28), R Studio (Version 4.1.2), and GraphPad Prism 9. Statistical significance was considered at 2-sided *P* values of <0.05.

## RESULTS

### Patients

An overview of patient enrollment is presented in **Figure S1** (**SDC**, http://links.lww.com/TP/D91). In total, we included 125 patients for analysis. There were no major differences in patient characteristics or in SARS-CoV-2-specific immune responses at baseline between MMF/MPA continuation and discontinuation group, between 1× mRNA-1273 and 2× mRNA-1273 group and between 1× mRNA-1273 and Ad26.COV2.S group (Table 1; **Table S1**, **SDC**, http://links.lww.com/TP/D91). Baseline characteristics of the control groups, consisting of healthy individuals and KTR receiving 2 primary mRNA-1273 vaccinations, have been described previously.^8^ There were no significant differences in baseline characteristics between these 2 control groups.

**TABLE 1.** Baseline characteristics of patients included for IL-21 and B-cell ELISpot

	Mycophenolate mofetil—mycophenolic acid discontinuation study group		Alternative vaccination study group		
	MMF+ (n = 25)	MMF− (n = 25)	1× mRNA-1273 (n = 25)	2× mRNA-1273 (n = 25)	Ad26.COV2-S (n = 25)
Age, y	61 (53–71)	63 (54–70)	62 (52–68)	63 (55–73)	60 (46–68)
Female	13 (52)	9 (36)	6 (24)	12 (48)	8 (32)
Ethnicity					
White	25 (100)	24 (96)	24 (96)	24 (96)	22 (88)
Asian	0	1 (4)	1 (4)	0	2 (8)
Black	0	0	0	0	1 (4)
Other	0	0	0	1 (4)	0
Body mass index, kg/m^2^	26 (22–30)	27 (25–30)	26 (23–27)	24 (23–28)	25 (22–31)
Comorbidities					
Hypertension	18 (72)	20 (80)	23 (92)	19 (76)	21 (84)
Diabetes	6 (24)	5 (20)	12 (48)	7 (28)	2 (8)
History of coronary artery disease	4 (16)	3 (12)	4 (16)	2 (8)	3 (12)
Heart failure	1 (4)	1 (4)	0	0	0
Chronic lung disease	2 (8)	1 (4)	0	2 (8)	3 (12)
History of malignancy^*a*^	2 (8)	7 (28)	2 (8)	6 (24)	1 (4)
Autoimmune disease	4 (16)	2 (8)	0	1 (4)	1 (4)
Lymphocytes, 10^9^/L	1.5 (1.1–1.6)	1.3 (1.0–1.7)	1.4 (1.1–2.0)	1.3 (0.9–2.0)	1.1 (0.8–1.6)
eGFR, mL/min/1.73 m^2^	49.7 (15.9)	51.5 (19.7)	47.1 (18.1)	53.4 (21.7)	50.4 (22.6)
Primary renal diagnosis					
Primary glomerulonephritis	5 (24)	2 (10)	3 (14)	4 (18)	3 (14)
Pyelonephritis	0	0	1 (5)	3 (14)	0
Interstitial nephritis	0	1 (5)	0	1 (5)	4 (19)
Familial-hereditary renal disease	2 (10)	4 (20)	4 (19)	8 (36)	3 (14)
Congenital diseases	1 (5)	1 (5)	1 (5)	1 (5)	2 (10)
Vascular diseases	2 (10)	2 (10)	2 (10)	0	3 (14)
Secondary glomerular systemic disease	0	0	3 (14)	4 (18)	4 (19)
Diabetic kidney disease	1 (5)	2 (10)	5 (24)	0	0
Other	10 (48)	8 (40)	2 (10)	1 (5)	2 (10)
Transplant characteristics					
First kidney transplant	23 (92)	23 (92)	24 (96)	17 (68)	21 (84)
Time after last transplantation, y	5.0 (2.0–8.0)	4.0 (1.0–6.5)	5.0 (2.5–11.5)	10.0 (2.5–13.5)	9.5 (2.0–17.0)
Last transplant					
Living donor	21 (84)	15 (60)	15 (60)	20 (80)	18 (72)
Deceased donor	4 (16)	10 (40)	10 (40)	5 (20)	7 (28)
Immunosuppressive treatment					
Steroids	25 (100)	25 (100)	16 (64)	14 (56)	16 (64)
Azathioprine	0	0	0	1 (4)	1 (4)
Mycophenolate mofetil or mycophenolic acid	25 (100)	25 (100)	23 (92)	20 (80)	18 (72)
Calcineurin inhibitor	25 (100)	25 (100)	21 (84)	21 (84)	18 (72)
mTOR inhibitor	0	0	0	1 (4)	0
Other	0	1 (4)	0	1	0
No. of previous SARS-CoV-2 vaccinations					
2	20 (80)	14 (56)	25 (100)	25 (100)	25 (100)
3	5 (20)	11 (44)	0	0	0
Time since last SARS-CoV-2 vaccination	180 (167–193)	179 (103–205)	198 (192–206)	198 (187–222)	198 (195–199)

Data are n (%), mean (SD), or median (IQR).

^*a*^ Including melanomas, excluding all other skin malignancies.

Ad26.COV2.S, adenovirus type 26 encoding the SARS-CoV-2 spike glycoprotein; eGFR, estimated glomerular filtration rate; ELISpot, enzyme-linked immunosorbent spot; IL-21, interleukin-21; IQR, interquartile range; MMF, mycophenolate mofetil; mRNA-1273, messenger RNA-1273; mTOR, mammalian target of rapamycin.

### Vaccine-induced SARS-CoV-2-specific Memory T-cell Responses

**FIGURE 1.**
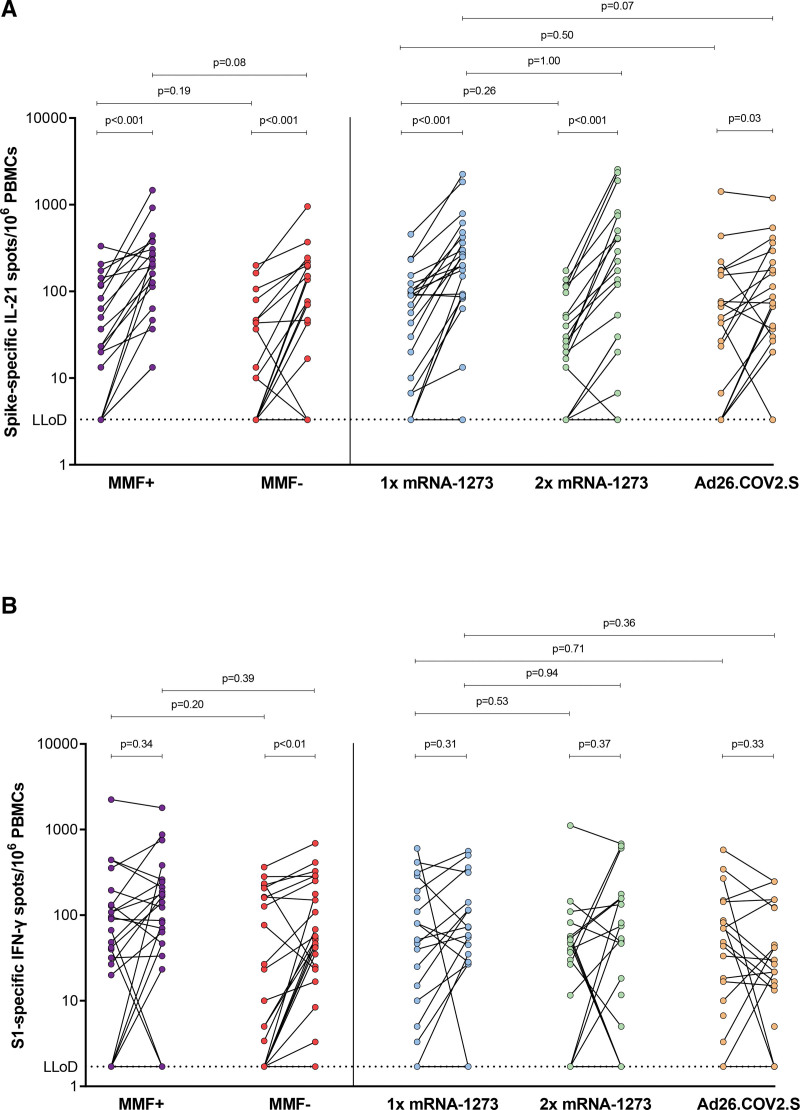
SARS-CoV-2-specific T-cell responses. A, IL-21 memory T-cell response. B, IFN-γ response. The MMF+ group continued and the MMF− group discontinued mycophenolate mofetil/mycophenolic acid treatment 1 wk before and 1 wk after receiving repeated vaccination with 1 dose (100 μg) of mRNA-1273. Data are presented as paired data. Mann-Whitney *U* test (pairwise deletion) or Wilcoxon singed-rank test (listwise deletion) was used to compare medians. The LLoD response was 3.3 spots for IL-21 memory T-cell response and 1.7 spots for IFN-γ response. Each symbol represents an individual. Number of missing cases (of total 125) is 23 for IL-21 and 18 for IFN-γ. Ad26.COV2.S, adenovirus type 26 encoding the SARS-CoV-2 spike glycoprotein; IFN-γ, interferon gamma; IL-21, interleukin-21; LLoD, lower limit of detection; MMF, mycophenolate mofetil; mRNA-1273, messenger RNA-1273; PBMCs, peripheral blood mononuclear cells; S1, spike protein.

To determine the memory T-cell response, SARS-CoV-2-specific IL-21–producing memory T cells were assessed in 110 patients at baseline and 107 patients 28 d post-repeated vaccination, providing paired data for 102 patients (**Figure S1**, **SDC**, http://links.lww.com/TP/D91). Missing data were because of a lack of PBMCs or technical failure. All vaccination groups showed significantly increased SARS-CoV-2-specific IL-21 memory T-cell responses compared with baseline (Figure 1A). At baseline, 74% (75/102) of participants showed detectable SARS-CoV-2-specific IL-21–producing memory T cells (>LLoD), despite having poor serological responses (**Figure S2A**, **SDC**, http://links.lww.com/TP/D91). After repeated vaccination, a significant increase in IL-21–producing T cells was observed in 93% (95/102) of the patients (**Figure S2B**, **SDC**, http://links.lww.com/TP/D91). IL-21 memory T-cell response was comparable between the groups, both at baseline and day 28 after repeated vaccination (Figure 1A). Similarly, no differences were observed in the fold changes between groups (**Figure S3A**, **SDC**, http://links.lww.com/TP/D91). The positive control (PBMCs stimulated with phytohaemagglutinin) showed no significant differences between the various cohorts (**Figure S4A**, **SDC**, http://links.lww.com/TP/D91).

Next, we compared IFN-γ and IL-21–producing T-cell responses in KTR following repeated vaccination. These data show that, unlike IL-21, SARS-CoV-2 spike-specific IFN-γ responses increased in none of the vaccination groups, except for the MMF− group (*P* < 0.01) (Figure 1B).^6^ At baseline, 74% (79/107) of patients already showed detectable IFN-γ producing T cells (>LLoD), despite being poor serological responders (**Figure S2A**, **SDC**, http://links.lww.com/TP/D91). After repeated vaccination, this further increased to 82% (88/107) of patients across all groups (**Figure S2B**, **SDC**, http://links.lww.com/TP/D91). Both IL-21 and IFN-γ were positively correlated at baseline (Spearman’s ρ = 0.3, *P* < 0.01; **Figure S5A**, **SDC**, http://links.lww.com/TP/D91) and at 28 d after repeated vaccination (Spearman’s ρ = 0.4, *P* < 0.001; **Figure S5B**, **SDC**, http://links.lww.com/TP/D91).

### Vaccine-induced SARS-CoV-2-specific Memory B-cell and Antibody Responses

**FIGURE 2.**
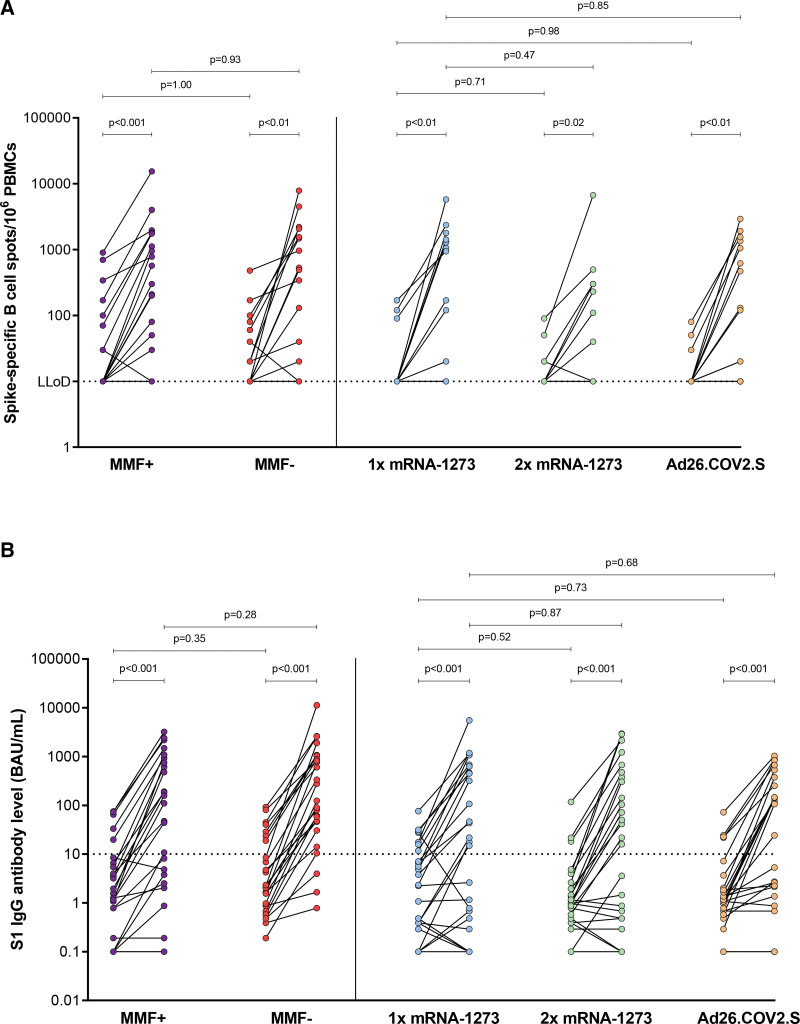
Vaccine-induced SARS-CoV-2-specific B-cell–mediated immune responses. A, Memory B-cell response. B, Antibody response. The MMF+ group continued and the MMF− group discontinued mycophenolate mofetil/mycophenolic acid treatment 1 wk before and 1 wk after receiving repeated vaccination with 1 dose (100 μg) of mRNA-1273. Data are presented as paired data. Mann-Whitney *U* test (pairwise deletion) or Wilcoxon singed-rank test (listwise deletion) was used to compare medians. The LLoD response was 10 spots for memory B-cell response. Dotted line in B represents the cutoff value for being a serological responder (≥10 BAU/mL). Each symbol represents an individual. Number of missing cases (of total 125) = 35 for memory B-cell response. Ad26.COV2.S, adenovirus type 26 encoding the SARS-CoV-2 spike glycoprotein; BAU, binding antibody units; LLoD, lower limit of detection; MMF, mycophenolate mofetil; mRNA-1273, messenger RNA-1273; PBMCs, peripheral blood mononuclear cells; S1, spike protein.

To assess the memory B-cell response, SARS-CoV-2-specific IgG producing memory B cells were measured in 97 patients at baseline and 104 patients 28 d post-repeated vaccination, with paired data available for 90 patients (**Figure S1**, **SDC**, http://links.lww.com/TP/D91). All alternative vaccination strategies resulted in increased SARS-CoV-2-specific memory B-cell responses (Figure 2A). At baseline, 30% (27/90) of patients exhibited detectable SARS-CoV-2-specific IgG producing memory B cells (>LLoD) (**Figure S2C**, **SDC**, http://links.lww.com/TP/D91), which increased to 64% (58/90) post-vaccination (**Figure S2D**, **SDC**, http://links.lww.com/TP/D91). The fold change did not differ between groups (**Figure S3B**, **SDC**, http://links.lww.com/TP/D91). The positive control (total IgG producing cells) also showed no significant differences between the cohorts (**Figure S4B**, **SDC**, http://links.lww.com/TP/D91). The median spike S1-specific antibody titer at baseline was 1.36 BAU/mL (0.58–6.73 BAU/mL) for all patients in this substudy, indicating a poor serological status before repeated vaccination (**Table S1**, **SDC**, http://links.lww.com/TP/D91).^6^ Consistent with the data from the primary study, all groups showed a significant increase in SARS-CoV-2 spike S1-specific antibodies after repeated vaccination, with no superiority of a higher dose, a heterologous vaccine, or 2 wk discontinuation of MMF/MPA (Figure 2B).^6^

### Correlations Between SARS-CoV-2-specific Memory T- and B-cell Responses

**FIGURE 3.**
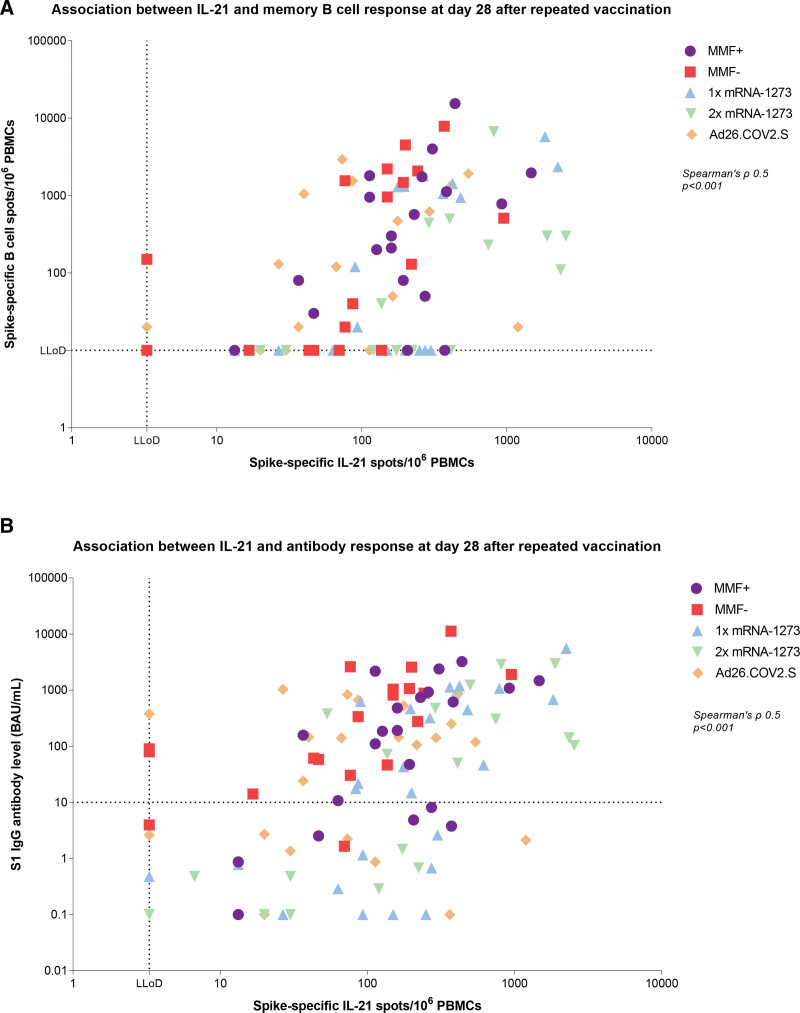
Correlations between IL-21 memory T-cell and B-cell–mediated immune responses at 28 d after repeated vaccination. A, Correlation IL-21 and memory B-cell response. B, Correlation IL-21 and antibody response. The MMF+ group continued and the MMF− group discontinued mycophenolate mofetil/mycophenolic acid treatment 1 wk before and 1 wk after receiving repeated vaccination with 1 dose (100 μg) of mRNA-1273. The horizontal dotted line represents the LLoD of memory B-cell response (=10 spots) or the cutoff value for being a serological responder (≥10 BAU/mL) and the vertical dotted line represents the LLoD of IL-21 memory T-cell response (=3.3 spots). Ad26.COV2.S, adenovirus type 26 encoding the SARS-CoV-2 spike glycoprotein; BAU, binding antibody units; IL-21, interleukin-21; LLoD, lower limit of detection; MMF, mycophenolate mofetil; mRNA-1273, messenger RNA-1273; PBMCs, peripheral blood mononuclear cells; S1, spike protein.

**FIGURE 4.**
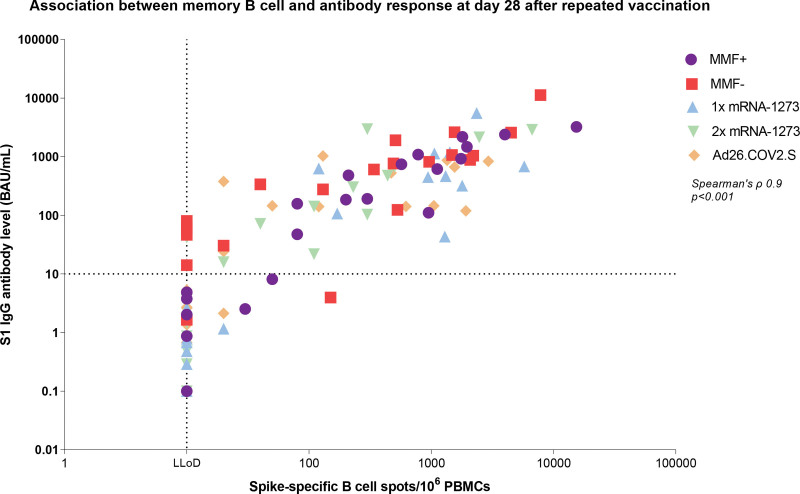
Correlation between SARS-CoV-2-specific memory B-cell response and SARS-CoV-2 spike S1-specific IgG antibody level at 28 d after repeated vaccination. The MMF+ group continued and the MMF− group discontinued mycophenolate mofetil/mycophenolic acid treatment 1 wk before and 1 wk after receiving repeated vaccination with 1 dose (100 μg) of mRNA-1273. The horizontal dotted line represents the cutoff value for being a serological responder (≥10 BAU/mL) and the vertical dotted line represents the LLoD of memory B-cell response (=10 spots). Ad26.COV2.S, adenovirus type 26 encoding the SARS-CoV-2 spike glycoprotein; BAU, binding antibody units; LLoD, lower limit of detection; MMF, mycophenolate mofetil; mRNA-1273, messenger RNA-1273; PBMCs, peripheral blood mononuclear cells; S1, spike protein.

In this total patient cohort, positive associations were observed at 28 d after repeated vaccination among all patients: between IL-21 memory T-cell and memory B-cell response (Spearman’s ρ = 0.5, *P* < 0.001; Figure 3A), between IFN-γ memory T-cell and memory B-cell response (Spearman’s ρ = 0.3, *P* < 0.01; **Figure S5C**, **SDC**, http://links.lww.com/TP/D91), and between memory T-cell IL-21 and antibody response (Spearman’s ρ = 0.5, *P* < 0.001; Figure 3B) and IFN-γ memory T-cell and antibody response (Spearman’s ρ = 0.3, *P* < 0.001; **Figure S5D**, **SDC**, http://links.lww.com/TP/D91). Of note, 30% (28/92) of patients who showed detectable IL-21–producing T cells lacked detectable memory B cells at 28 d after repeated vaccination (Figure 3A). Similarly, 31% (30/99) exhibited an IL-21 memory T-cell response without seroconversion (Figure 3B). A strong positive correlation was found between SARS-CoV-2-specific memory B-cell response and SARS-CoV-2 spike S1-specific IgG antibody level at 28 d after repeated vaccination across all patients (Spearman’s ρ = 0.9, *P* < 0.001; Figure 4).

### Analysis of Cellular Activity in Seroconversion Compared With Controls

Our previous study showed a seroconversion rate of 100% in healthy individuals and 65% in KTR (41/63) after receiving 2 primary mRNA-1273 vaccinations.^8^ KTR in this study were selected based on a poor humoral response to a second or third mRNA-based vaccination with 21% (26/125) of these individuals being seropositive at baseline. Following repeated vaccination, this proportion increased to 66% (83/125) (**Table S1**, **SDC**, http://links.lww.com/TP/D91). The observed heterogeneity in seroconversion following vaccination suggests potential differences in cellular activity between these groups, which were investigated by categorizing correlation graphs. Additionally, the potential of T and B cells to classify seroconversion was explored by generating receiver operating characteristic curves and calculating corresponding AUCs.

**FIGURE 5.**
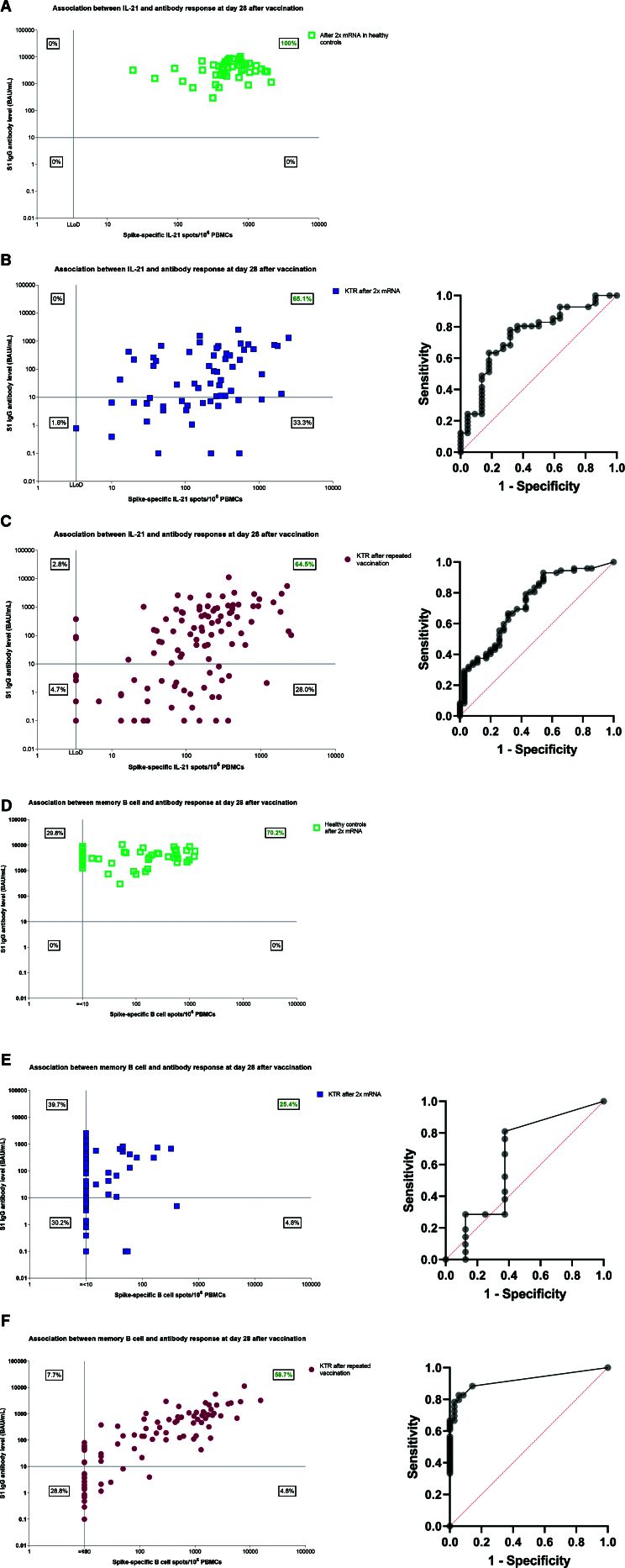
Association between cellular memory activity and seroconversion. Correlation between SARS-CoV-2-specific IL-21 memory T-cell and antibody level at 28 d after the second vaccination in healthy individuals (A) and in KTRs (B), and at 28 d after repeated vaccination in KTR (C). Correlation between SARS-CoV-2-specific memory B-cell and antibody level at 28 d after the second vaccination in healthy individuals (D) and in KTRs (E), and at 28 d after repeated vaccination in KTR (F). The horizontal line represents the cutoff value for being a serological responder (≥10 BAU/mL), and the vertical line represents the LLoD of the IL-21 memory T-cell (=3.3 spots) or memory B-cell response (≤10 spots). B, C, E, and F, Receiver operating characteristic (ROC) curves are presented using IL-21 or memory B-cell response as continuous variable and seroconversion (yes or no) as a binary outcome. The number of IL-21–producing memory T cells showed potential for classifying seroconversion in KTR after both 2 primary vaccinations (B; AUC, 0.75; 95% CI, 0.62-0.88; *P* < 0.01) and repeated vaccination (C; AUC, 0.73; 95% CI, 0.63-0.83; *P* < 0.01). The number of antigen-specific memory B cells did not show potential for classifying seroconversion in KTR after 2 primary vaccinations (E; AUC, 0.64; 95% CI, 0.37-0.90; *P* = 0.26), but did after repeated vaccination (F; AUC, 0.92; 95% CI, 0.87-0.97; P < 0.0001). AUC, area under the curve; BAU, binding antibody units; CI, confidence interval; IL-21, interleukin-21; KTR, kidney transplant recipient; LLoD, lower limit of detection; mRNA, messenger RNA; PBMCs, peripheral blood mononuclear cells; S1, spike protein.

Our analyses revealed that the association between IL-21 memory T cells and seroconversion is weaker in KTR compared with healthy individuals. Among healthy individuals who received 2 primary vaccinations, a IL-21 memory T-cell response was consistently associated with seroconversion in all cases (Figure 5A).^8^ However, among KTR receiving 2 primary vaccinations, only 65% of patients with a memory T-cell response seroconverted (Figure 5B).^8^ This association was similarly observed in KTR who received repeated vaccination, where 65% of patients with a memory T-cell response were also serological responders (Figure 5C). Although a memory T-cell response did not always result in the induction of virus-specific antibodies in KTR, the presence of IL-21–producing memory T cells was associated with seroconversion following both 2 primary vaccinations (OR, 4.66 [1.70-12.77]; *P* < 0.01) and repeated vaccination (OR, 3.84 [1.89-7.78]; *P* < 0.001). We demonstrated that the number of IL-21–producing memory T cells showed potential for classifying seroconversion in patients after both 2 primary vaccinations (Figure 5B; AUC, 0.75; 95% CI, 0.62-0.88; *P* < 0.01) and repeated vaccination (Figure 5C; AUC, 0.73; 95% CI, 0.63-0.83; *P* < 0.01).

Regarding memory B-cell activity, we observed no association between antigen-specific memory B cells and seroconversion in KTR who received 2 primary vaccinations (Figure 5E). This is in contrast to both healthy individuals receiving 2 vaccinations (Figure 5D) and KTR who received repeated vaccination (Figure 5F). Among healthy individuals who received 2 primary vaccinations, 14 of 47 (30%) showed a memory B-cell response at the LLoD, while seroconversion was present in all cases 28 d after vaccination(Figure 5D).^8^ For KTR receiving 2 primary vaccinations, 44 of 63 (70%) had a memory B-cell response at the LLoD, with 25 of them also showing seroconversion (Figure 5E).^8^ In these patients with a memory B-cell response (>LLoD), 16 of 63 (25%) showed seroconversion (Figure 5E). As a result, memory B-cell activity was not found to be associated with seroconversion (OR, 2.40 [0.51-11.21]; *P* = 0.27), and the number of antigen-specific memory B cells did not show potential for classifying seroconversion in patients after 2 primary vaccinations (Figure 5E; AUC, 0.64; 95% CI, 0.37-0.90; *P* = 0.26). In contrast, among KTR who received repeated vaccination, the percentage of patients demonstrating both a memory B-cell response and seroconversion was 59% (Figure 5F). Consequently, the association between memory B-cell activity and seroconversion was found to be statistically significant (OR, 35.93 [6.94-186.04]; *P* < 0.001), and the number of antigen-specific memory B cells showed potential for classifying seroconversion in patients after repeated vaccination (Figure 5F; AUC, 0.92; 95% CI, 0.87-0.97; *P* < 0.0001).

## DISCUSSION

We explored cellular immune responses, in KTR who were previously defined as poor serological responders after 2 or 3 doses of an mRNA-based COVID-19 vaccine, and who were randomized to receive repeated vaccination with a double dose of an mRNA-1273 vaccine, heterologous vaccination with Ad26.COV2.S or temporary discontinuation of MMF/MPA compared with standard dose mRNA-1273 vaccination. We found no differences between groups in SARS-CoV-2-specific IL-21 memory T-cell and memory B-cell responses. In addition, we demonstrated that in KTR who received repeated vaccination, seroconversion was strongly associated with an increase in antigen-specific memory B cells, with the number of these cells showing potential for classifying seroconversion. This is in contrast to KTR who received 2 primary vaccinations, where we did not observe a similar association and found no potential for classifying seroconversion based on the number of B cells.

Here, we found that after a third or fourth COVID-19 vaccination, there was a significant increase in IL-21 memory T-cell and B-cell responses in KTR whose humoral response was impaired, although these responses remained lower than those observed in healthy individuals after 2 primary mRNA-1273 vaccinations.^8^ Although IFN-γ producing T cells represent the current “gold standard” for assessing cellular responses to COVID-19 vaccination, IFN-γ responses did not increase following repeated vaccination, in contrast to IL-21 responses. This suggests that IFN-γ may not be a sufficient biomarker for assessing T-cell memory, as it underestimates T-cell responses induced by COVID-19 vaccination in KTR. However, studies investigating T-cell responses after the third or fourth COVID-19 vaccination in KTR have primarily assessed this by measuring the IFN-γ response.^4,6,23-25^ Measuring multiple T-cell cytokines could provide additional insights into cellular immune responses and humoral immunity after repeated vaccination in KTR, as also shown by another study.^26^

We illustrated an association between seroconversion after repeated COVID-19 vaccination and the number of peripheral memory T and B cells, while this association was not observed for B cells following 2 primary vaccinations in KTR. This may indicate that the development of antigen-specific B cells in germinal centers and migration to the peripheral compartment is delayed or impaired as we quantified these cells in the peripheral blood. The use of immunosuppressive drugs could intervene in these processes. First, immunosuppressants may affect germinal center lymph node reactions where T cells interact with B cells to undergo class switch recombination, somatic hypermutation, and enhance antibody functions.^15,27-29^ Second, the use of MMF/MPA inhibits T- and B-cell proliferation and plasma cell formation.^30^ Our finding may explain data indicating that MMF/MPA has a strong association with an impaired serological response to COVID-19 vaccination in KTR.^1,31-33^ Moreover, it has been shown that MMF/MPA is the primary factor contributing to a delayed humoral response after COVID-19 vaccination in these patients.^34^ Also, MMF/MPA was associated with reduced absolute B-cell numbers and hampered humoral responses in KTR exposed to pathogens (eg, pneumococcal polysaccharide and tetanus toxoid).^35^ Multiple rounds of B-cell stimulation through repeated COVID-19 vaccination resulted in increased B-cell activation, proliferation, and differentiation into immunoglobulin-secreting plasma cells.^36^ Previous data indeed showed that seroconversion rates increase per vaccination dose in these patient cohorts.^3^

In line with our previous observation for humoral responses, we could not show a beneficial effect of temporary discontinuation of MMF/MPA on cellular immune responses.^6^ A possible explanation for this could be the relatively short discontinuation period of 2 wk, which was chosen to minimize the risk of graft rejection. Although effects of MMF/MPA on T and B cells can be reversible upon discontinuation, it may take some time for lymphocyte populations to return to baseline levels and regain full functionality.^37^ A study that stopped MMF/MPA for 5 wk in KTR who did not seroconvert after previous COVID-19 vaccinations, demonstrated increased antigen-specific B cells, plasmablasts, and in vivo activated virus-specific T cells with no enhancement on memory differentiation and cytokine production after repeated vaccination.^38^ In contrast, another study, with similar patients and discontinuation period, showed no effect on T-cell response, as measured by IFN-γ.^39^ However, these studies did not include a control group without MMF/MPA discontinuation and/or were not randomized.

A strength of our post hoc study is that it builds upon a multicenter randomized controlled trial, which enabled us to compare cellular immunity after 3 alternative vaccination regimens in KTR and to compare that to patients who received the standard vaccination strategy.^6^ Furthermore, we had reference data available from a previously reported primary vaccination study in healthy individuals and KTR. We investigated both memory T- and B-cell responses induced by repeated COVID-19 vaccination in KTR with impaired SARS-CoV-2-specific humoral responses. Additionally, we included 2 different biomarkers for T-cell activity to provide further insight into T-cell function after repeated vaccination in immunosuppressed KTR. To ensure that the cellular memory immune responses were solely vaccine-induced, we excluded patients with previous SARS-CoV-2 infection. Our findings are relevant to other patients using immunosuppressive drugs and may contribute to optimizing vaccination strategies against other viruses in these patient groups.

Our study also has several limitations. First, cellular memory immune responses were assessed at baseline and 28 d after repeated vaccination. Therefore, we cannot draw conclusions regarding the long-term development and durability of these responses, which would need further evaluation. Nonetheless, the finding that on average 72% of patients exhibited a SARS-CoV-2-specific IL-21 memory T-cell response 6 mo after the last vaccination with no COVID-19 in the meantime, underlines the durability of this response. This is supported by other data demonstrating the persistence of coronavirus-specific T cells.^40-42^ Second, further research is needed to investigate the protective role of these cellular memory immune responses on COVID-19-related clinical outcomes in patients with impaired humoral immunity. Third, asymptomatic cases may have gone undetected because of the absence of routine polymerase chain reaction screening. However, clinically relevant COVID-19 cases were identified with SARS-CoV-2-specific nucleocapsid antibodies and self-reported SARS-CoV-2 positive polymerase chain reaction tests. Fourth, we demonstrated that the number of peripheral memory T and B cells have the potential to predict seroconversion after repeated vaccination. However, the observational nature of this study limited the ability to work towards a prediction model. Fifth, there were missing values for IL-21 and B-cell responses because of the lack of PBMCs or technical failure. However, imputation was not performed in accordance with the convention of not imputing outcome variables.^43^ Lastly, this was a post hoc analysis in which we focused more on hypothesis generation than hypothesis testing. Therefore, *P* values should be interpreted cautiously.

In conclusion, in KTR with initially poor serological responses, repeated vaccination enhanced SARS-CoV-2-specific memory T- and B-cell responses, and antibody formation. A higher vaccine dose, a heterologous vaccine administration, or 2-wk discontinuation of MMF/MPA did not prove superior in increasing these responses. Our immune monitoring data indicate that seroconversion after repeated vaccination is strongly associated with the number of antigen-specific peripheral blood memory T and B cells. This emphasizes the importance of virus-specific memory T- and B-cell responses for comprehensive understanding of COVID-19 vaccine efficacy among KTR. These findings underscore the importance of repeated vaccination as a crucial strategy for overcoming the impaired immune responses to SARS-CoV-2.

### ACKNOWLEDGMENTS

The authors thank the RECOVAC collaborators: Alferso C. Abrahams, MD, PhD (Department of Nephrology and Hypertension, University Medical Center Utrecht, Utrecht, the Netherlands); Marije C. Baas, MD, PhD (Department of Nephrology, Radboud University Medical Center, Radboud Institute for Health Sciences, Nijmegen, the Netherlands); Pim Bouwmans, MD (Department of Internal Medicine, Division of Nephrology, Maastricht University Medical Center and CARIM School for Cardiovascular Disease, University of Maastricht, Maastricht, the Netherlands); Marc A. G. J. ten Dam MD, PhD (Dutch Registry RENINE, Nefrovisie, Utrecht, the Netherlands); Lennert Gommers, BSc (Department Viroscience, Erasmus Medical Center, Rotterdam, the Netherlands); Dorien Standaar (Renal Transplant Unit, Amsterdam UMC, University of Amsterdam, Amsterdam, the Netherlands); Marieke van der Heiden, PhD (Department of Medical Microbiology and Infection Prevention, University Medical Center Groningen, Groningen, the Netherlands); Yvonne M. R. Adema (Department of Internal Medicine, Division of Nephrology, University of Groningen, University Medical Center Groningen, Groningen, the Netherlands); Marieken J Boer-Verschragen (Department of Internal Medicine, Nephrology and Transplantation, Erasmus MC Transplant Institute, Erasmus Medical Center, Rotterdam, the Netherlands); Wouter B. Mattheussens, MSc (Department of Nephrology, Radboud University Medical Center, Radboud Institute for Health Sciences, Nijmegen, the Netherlands); Ria H. L. A. Philipsen (Department of Nephrology, Radboud University Medical Center, Radboud Institute for Health Sciences, Nijmegen, the Netherlands); Djenolan van Mourik, BSc (Department Viroscience, Erasmus Medical Center, Rotterdam, the Netherlands); Susanne Bogers, MSc (Department Viroscience, Erasmus Medical Center, Rotterdam, the Netherlands); Laura L. A. van Dijk (Department Viroscience, Erasmus Medical Center, Rotterdam, the Netherlands); Nynke Rots, PhD (Center for Infectious Disease Control, National Institute for Public Health and the Environment, Bilthoven, the Netherlands); Marc H. Hemmelder, MD, PhD (Department of Internal Medicine, Division of Nephrology, Maastricht University Medical Center and CARIM School for Cardiovascular Disease, University of Maastricht, Maastricht, the Netherlands); and Aiko P. J. de Vries, MD, PhD (Department of Nephrology, Leiden University Medical Center, Leiden, the Netherlands).

## Supplementary Material


