# Supplementary material for: Repeated COVID-19 Vaccination Drives Memory T- and B-cell Responses in Kidney Transplant Recipients: Results From a Multicenter Randomized Controlled Trial
Source: Transplantation. 2024 Nov 21;108(12):2420–33. doi: 10.1097/TP.0000000000005119 (PMC11581438; doi:10.1097/TP.0000000000005119)
Supplement: Supplementary file 1 [file tpa-108-2420-s001.pdf]

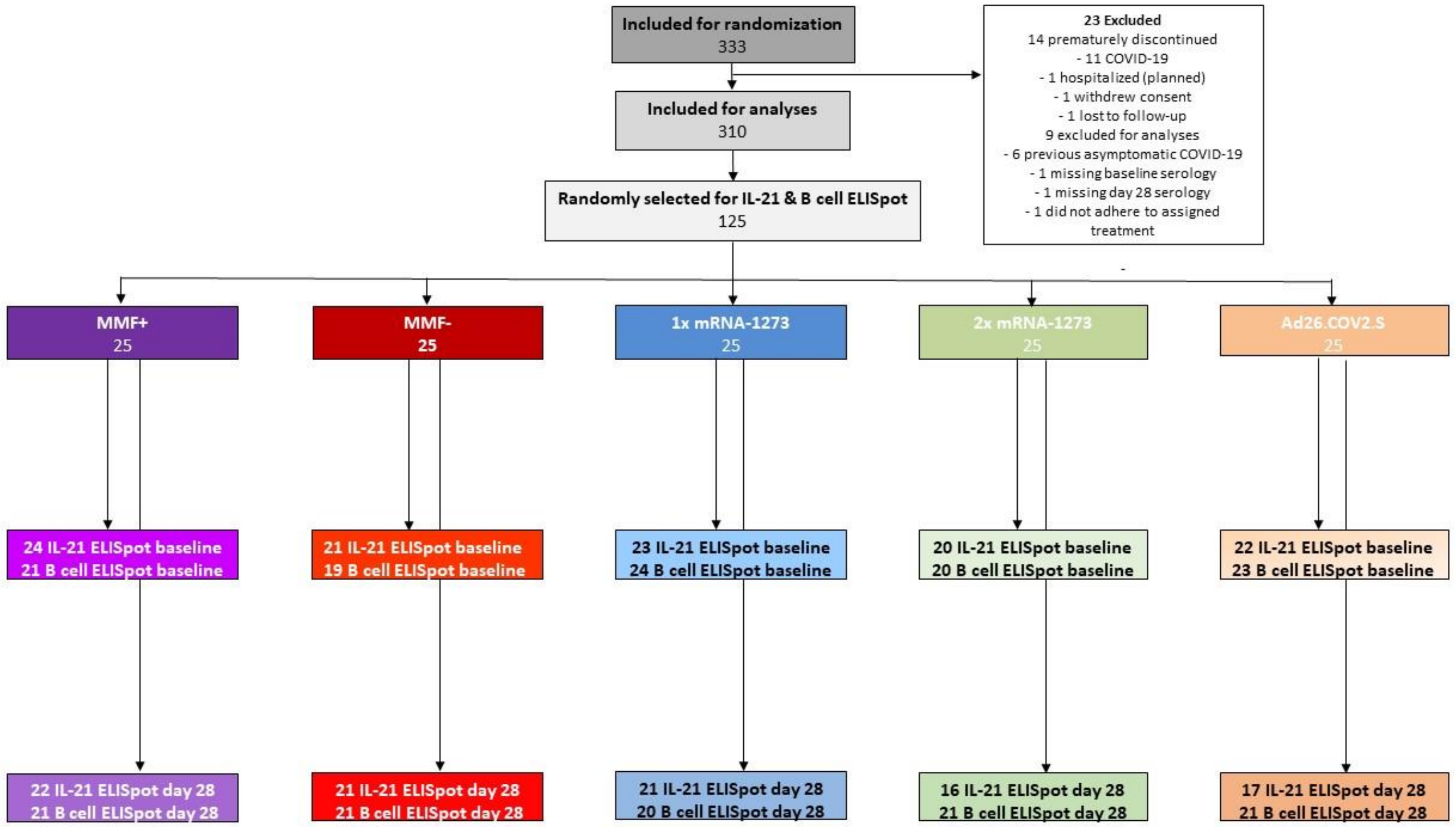

Figure S1. Flowchart of patient enrollment. The random selection of the 125 kidney transplant recipients from the original pool of study proceeded as follows: participants who had evidence of previous COVID-19 at baseline, indicated by nucleocapsid-specific antibodies (≥22 AU/mL) and Spike (S)1-specific antibodies (≥10 BAU/mL), or displaying exceptionally elevated antibody levels in S1, were excluded from the study. The selection process was stratified based on the randomization group. Patients lacking serology data at baseline and/or 28 days after repeated vaccination were excluded. Additionally, patients who developed COVID-19 before the visit of 28 days after repeated vaccination were also excluded, as most of these patients had no results at 28 days. Subsequently, random numbers were generated for each subject within each randomization group utilizing IBM SPSS statistics. Finally, the first 25 numbers from each study group were selected, resulting in a total of 125 kidney transplant recipients distributed evenly across the five distinct study groups.

Table S1. The vaccine-induced SARS-CoV-2-specific immune responses

	Mycophenolate mofetil - mycophenolic acid discontinuation study group		Alternative vaccination study group		
	MMF+ (n=25)	MMF- (n=25)	1 x mRNA-1273 (n=25)	2 x mRNA-1273 (n=25)	Ad26.COV2-S (n=25)
Immune response at baseline					
IL-21 memory T cell response	23.3 (3.3-100.0)	11.7 (3.3-86,7)	73.3 (6.7-145.8)	40.0 (13.3-113.3)	73.3 (3.3-176.7)
IFN-γ response	57.5 (6.3-130.9)	5.0 (1.7-161.7)	43.4 (2.9-166.7)	38.4 (1.7-56.7)	48.3 (10-143.3)
Memory B cell response	10 (10-85)	10 (10 -65)	10 (10-10)	10 (10-20)	10 (10-10)
Spike S1-specific antibodies	1.94 (0.49-14.10)	4.51 (1.48-31.61)	6.11 (0.63-25.03)	0.97 (0.39-2.62)	1.26 (1.07-7.17)
Serological responder[a]	5 (20)	7 (28)	7 (28)	3 (12)	4 (16)
Immune response at 28 days					
IL-21 memory T cell response	200.0 (113.3-356.7)	136.7 (58.3-210.0)	196.7 (76.7-393.3)	223.3 (30.0-813.3)	73.3 (28.3-235.0)
IFN-γ response	105.0 (36.7-235.4)	52.5 (23.7-233.0)	55.0 (14.2-128.3)	50.0 (1.7-157.5)	26.7 (14.2-122.5)
Memory B cell response	255 (35-1593)	130 (10-1815)	20 (10-1300)	10 (10-300)	120 (15-1200)

Spike S1-specific antibodies	187.89 (5.67-1045.73)	336.68 (52.53-1487.26)	2.62 (0.39-655.05)	50.01 (0.48-302.98)	141.21 (2.42-598.28)
Serological responder[a]	16 (64)	22 (88)	15 (60)	15 (60)	15 (60)

[a]Serological responder was defined as S1-specific IgG $\geq$10 BAU/mL.

Data are n (%) or median (IQR).

Lower limit of detection: 3.33 spots for IL-21, 10 spots for B cell, 1.7 spots for IFN-γ and 0.10 BAU/mL for antibody response.

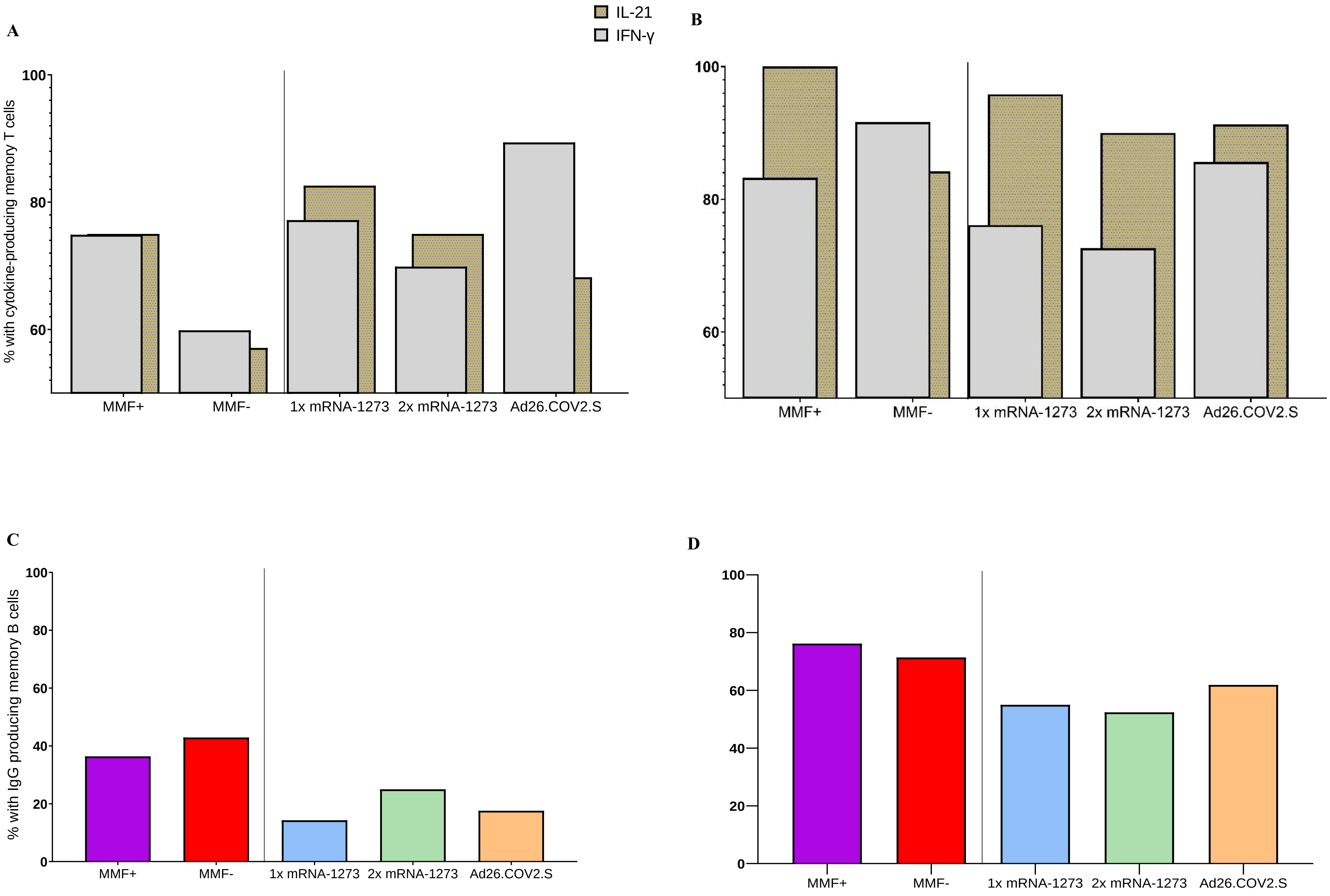
A
IL-21
IFN-γ
B
% with cytokine-producing memory T cells
100
80
60
MMF+
MMF-
1x mRNA-1273
2x mRNA-1273
Ad26.COV2.S
C
D
% with IgG producing memory B cells
100
80
60
40
20
0

Figure S2. Cytokine-producing memory T cells and IgG producing memory B cells. Data are presented as bar charts. Percentage of patients with IL-21 producing (=gold colored) or IFN-y producing (=grey colored) memory T cells at baseline (A) and at 28 days after repeated vaccination (B) per patient group. Percentage of patients with IgG producing memory B cells at baseline (C) and at 28 days after repeated vaccination (D) per patient group. The MMF+ group continued and the MMF- group discontinued mycophenolate mofetil/mycophenolic acid treatment one week before and one week after receiving repeated vaccination with one dose (100 µg) of mRNA-1273.

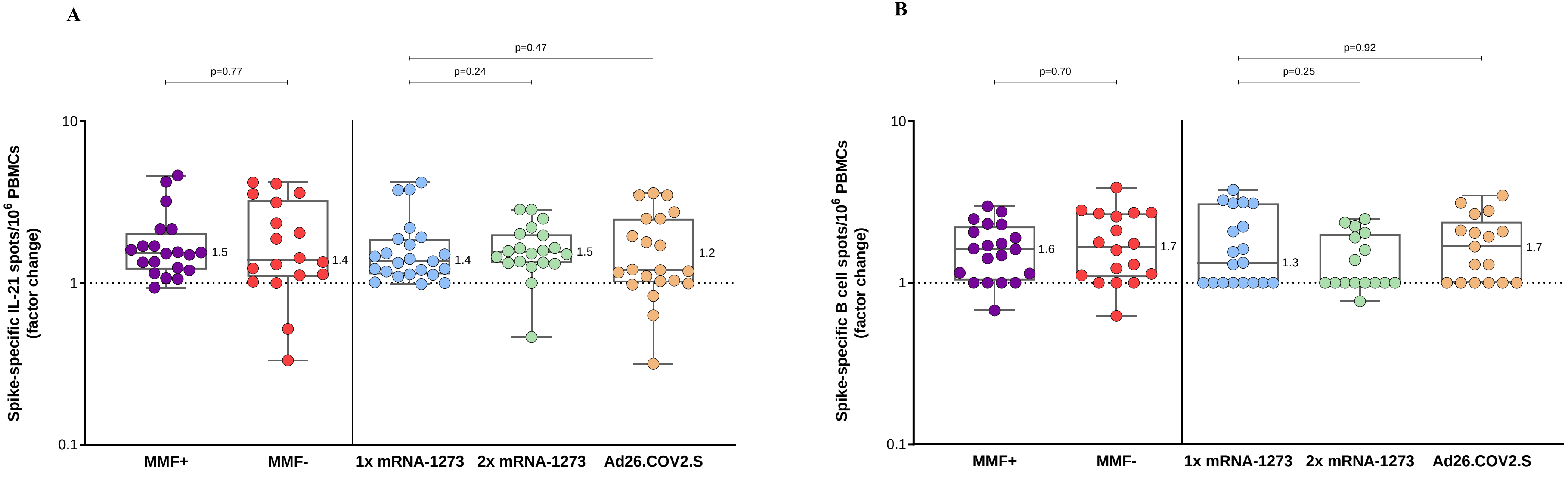

Figure S3. Fold change per patient. Data are presented in box-and-whisker plots. The MMF+ group continued and the MMF- group discontinued mycophenolate mofetil/mycophenolic acid treatment one week before and one week after receiving repeated vaccination with one dose (100 µg) of mRNA-1273. The horizontal line and numbers within the whisker indicate the medians and the tops and bottoms indicate interquartile ranges. Mann-Whitney U tests were applied to compare medians. Each symbol represents an individual. Fold change was calculated as the log-transformed response at 28 days after repeated vaccination divided by the log-transformed baseline response (factor change of 1 means neither an increase nor a decrease in response). A) Fold change for SARS-CoV-2-specific IL-21 memory T cell response. B) Fold change for SARS-CoV-2-specific memory B cell response.

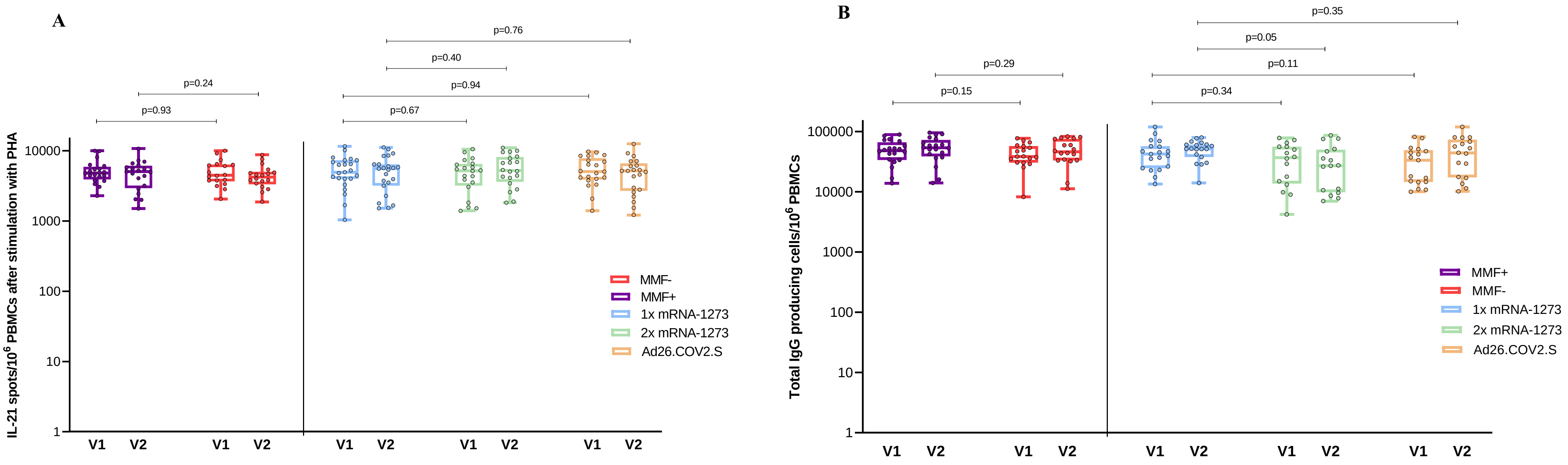

Figure S4. Positive controls for the IL-21 and B cell ELISpot assay. The MMF+ group continued and the MMF- group discontinued mycophenolate mofetil/mycophenolic acid treatment one week before and one week after receiving repeated vaccination with one dose (100 µg) of mRNA-1273. V1 stands for baseline and V2 stands for 28 days after repeated vaccination. A) The number of IL-21 spots per 1 million PBMCs/well at baseline and at 28 days after repeated vaccination per patient group after PBMCs were stimulated polyclonally with 1 µg/mL phytohaemagglutinin (PHA). B) The total IgG producing cells per 1 million PBMCs/well at baseline and at 28 days after repeated vaccination per patient group. Data are presented in box-and-whisker plots. The horizontal line and numbers within the whisker indicate the medians and the tops and bottoms indicate interquartile ranges. Mann-Whitney U tests were applied to compare medians.

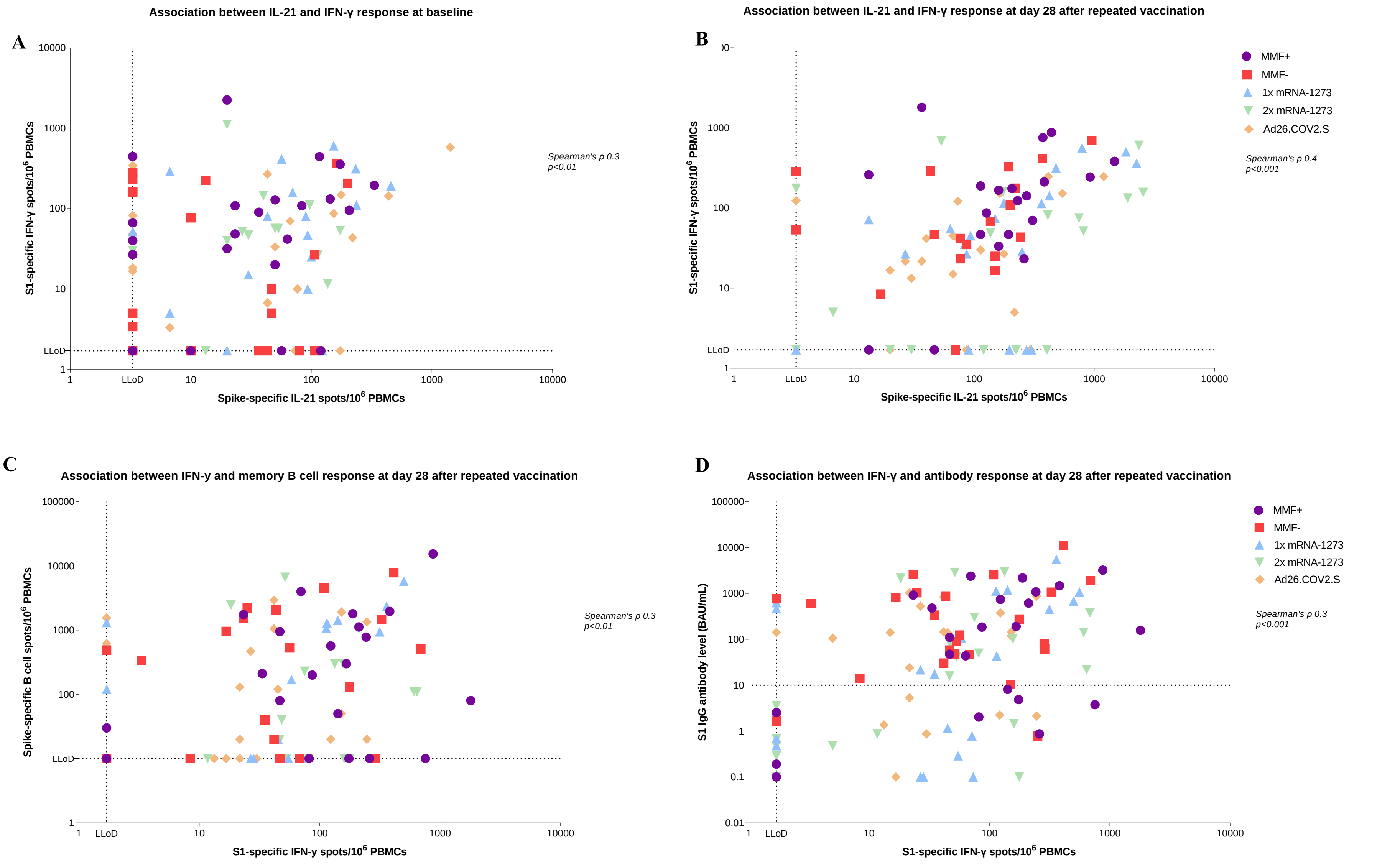

Figure S5. Correlations between SARS-CoV-2-specific IFN-γ response and other vaccine-induced immune responses. Correlation between IL-21 and IFN-γ response at baseline (A) and at 28 days after repeated vaccination (B). Correlation between IFN-γ response and memory B cell (C) and antibody responses (D) at 28 days after repeated

vaccination. The MMF+ group continued and the MMF- group discontinued mycophenolate mofetil/mycophenolic acid treatment one week before and one week after receiving repeated vaccination with one dose (100 µg) of mRNA-1273. The horizontal dotted line represents the lower limit of detection of IFN-γ (=1.7 spots), memory B cell response (=10 spots) or the cut-off value for being a serological responder (≥10 BAU/mL). The vertical dotted line represents the lower limit of detection of IL-21 memory T cell response (=3.3 spots) or IFN-γ response (=1.7 spots).
